# Genome-wide studies of *PAL* genes in sorghum and their responses to aphid infestation

**DOI:** 10.1038/s41598-022-25214-1

**Published:** 2022-12-29

**Authors:** Shankar Pant, Yinghua Huang

**Affiliations:** United States Department of Agriculture - Agricultural Research Service (USDA-ARS), Plant Science Research Laboratory, Stillwater, OK 74075 USA

**Keywords:** Genetics, Plant sciences

## Abstract

Phenylalanine ammonia-lyase (PAL, EC 4.3.1.25) plays a crucial role in plant adaptation to biotic and abiotic stresses. However, the current knowledge about PAL proteins in sorghum is essentially lacking. Thus, in this study we aimed to analyze the *PAL* family genes in sorghum using a genome-wide approach and to explore the role of *PAL* genes in host plant resistance to aphids via SA-mediated defense signaling. Here, we report gene structural features of 8 *PAL* (*SbPAL*) genes in sorghum (*Sorghum bicolor*), their phylogeny, protein motifs and promoter analysis. Furthermore, we demonstrated that the *SbPAL* genes were induced by sugarcane aphid (SCA) infestation and *SbPAL* exhibited differential gene expression in susceptible and resistant genotypes. PAL activity assays further validated upregulated expression of the *SbPAL* genes in a resistant genotype. In addition, exogenous application of SA reduced plant damage and suppressed aphid population growth and fecundity in susceptible genotype, suggesting that those *SbPAL* genes act as positive regulator of the SA-mediated defense signaling pathway to combat aphid pests in sorghum. This study provides insights for further examination of the defense role of PAL in sorghum against other pests and pathogens.

## Introduction

Phenylalanine ammonia-lyase (PAL) is an important enzyme of phenylpropanoid pathway that catalyzes non-oxidative deamination of L-phenylalanine into trans-cinnamate. This is the first key rate-limiting enzyme of the phenylpropanoid pathway which links primary metabolism with secondary metabolism. The pathway is rich in phenylpropanoid compounds, which are precursors to a wide range of phenolic compounds, such as flavonoids, plant hormones, anthocyanins, lignin, and phytoalexins^1,2^. Phenylpropanoid derivatives play an important role in plant defense against pests and pathogens. They respond to various abiotic stresses (UV lights, low temperature, nutrient stress), serve as regulatory molecules, and are involved in signal transduction and communication with other organisms^1,3–6^. Phenylpropanoid compounds are also utilized in lignin biosynthesis which confers stem rigidity, vascular integrity, and serve as a physical barrier to invading pathogens in plants^5^.

Several studies have shown that PALs in dicots are monofunctional in that they can only utilize phenylalanine as a substrate (PAL activity), whereas some monocot PALs are bifunctional, utilize phenylalanine and tyrosine as substrates (PAL and TAL activity)^7–9^. PAL is encoded by a multigene family, and their expression in response to biotic and abiotic stresses is highly regulated, temporally and spatially^10^. Several studies state that increased PAL activity is associated with the earliest responses of plant to pathogens^3,11^. Since PAL is involved in biosynthesis of several phenylpropanoid compounds with defense properties, an elevated PAL level is usually considered as an indicator of resistance in plants^12^. It is well established that PAL is involved in salicylic acid (SA) biosynthesis and induced PAL expression is related with SA accumulation in several plants^13–16^. The defense role of PAL and SA to pathogens have been demonstrated in several studies via *PAL*-silencing and exogenous application of SA experiments in Arabidopsis, tobacco, Brachypodoium, *Lotus japonicum*, maize and pepper^13,14,17–24^. However, SA biosynthesis pathways have been determined only in few plants. Two pathways of SA biosynthesis in plants have been identified, chorismate and phenylpropanoid pathways, which are regulated by isochorismate synthase (ICS) and PAL, respectively^20,25,26^. In Arabidopsis and barley, ICS is the dominant pathway for SA biosynthesis^25^. In tobacco, a majority of SA is synthesized via the phenylpropanoid pathway^27^, whereas SA synthesis in soybean is equally contributed by both pathways^15^. Currently, it is unclear which pathway is dominant in sorghum for SA biosynthesis.

Sorghum [*Sorghum bicolor* (L) Moench] is an important food, feed and biofuel crop with over a $2 billion economic impact in the United States^28^. Sorghum is well-adapted to several abiotic stresses such as high temperature and dry weather. However, pests and pathogens remain a major challenge to enhance sorghum production. Various insect pests feed on sorghum from seedling to mature stages. Among them, sugarcane aphid [*Melanaphis sacchari* (Zehntner)] is a major pest of sorghum and was first reported in 1922 in the United States^29^. Sugarcane aphid (SCA) was a sporadic pest of sugarcane until recently, when a major outbreak occurred on sorghum in 2013 in Texas and has since become a perennial pest affecting sorghum production in more than 20 states in the United States^30,31^. Besides being a vector for the sugarcane yellow leaf virus, SCA also possess potential threats to sugarcane production in the U.S. and elsewhere^32^. Although there are some resistant varieties of sorghum available against SCA^33–35^, however the mechanism of resistance is yet to be explored.

Understanding the defense mechanisms and genes involved in host plant resistance is necessary for improving sorghum crop by incorporating these resistance genes into new lines. However, there are limited literatures in identification and characterization of the functional role of potential resistance genes, which has hampered the sorghum breeding efforts for SCA resistance. Characterization of the potential SCA resistance genes of sorghum will improve our understanding of plant defense against SCA in resistant hybrids and cultivars. Thus, we aimed to study whether *SbPAL* genes have any role in host-mediated defense response during SCA infestation. The present study was carried out with the following two objectives: (1) to characterize *SbPAL* gene families in sorghum using a genome-wide approach and (2) to elaborate protective role of *SbPAL* in sorghum plant resistance to sugarcane aphids.

## Materials and methods

### Plant materials and growth conditions

Two varieties of sorghum (*Sorghum bicolor*) with varied responses to sugarcane aphid, Tx7000 susceptible and Tx2783 resistant to sugarcane aphid, were available to public use, which were originally obtained from the Germplasm Resources Information Network (GRIN, https://www.ars-grin.gov) in the U.S.; thus were selected for this experiment. All plant materials used in this study comply with local and national guidelines. Seeds were planted in plastic pots (7.5 cm in diameter; 7.1 cm in height), containing potting compost (Sungro Professional Growing Mix, Agawam, MA) after treating with fungicide (Captan 50W, Bonham, TX) and watered alternating days. Plants were grown in greenhouse set at constant temperature (28 +/− 2 °C) with a 14/10 (light/dark) photoperiod and 60% relative humidity.

### Sugarcane aphid rearing and plant infestation

Sugarcane aphid (SCA) were maintained for generations on Tx7000 plants in greenhouse conditions as described previously^24,36^. Three leaf stage sorghum seedlings were infested with similar-size 20 adults apterous SCA on adaxial surface of the first leaf. Plants were covered individually with cylindrical cages (SABIC Polymershapes, Tulsa, OK) to avoid SCA escape and unwanted pathogen/pest infestation. Plants without SCA infestation were also kept inside a cage as control.

### Sequence acquisition and phylogenetic analysis of PAL genes

We obtained the SbPAL amino acid sequence of *Sorghum bicolor* (sorghum), *Brachypodium distachyon* (*Brachypodium*) and *Arabidopsis thaliana* (Arabidopsis) from phytozome (https://phytozome.jgi.doe.gov/pz/portal.html). Amino acid sequences of *Oryza sativa* Japonica Group (rice) and *Zea mays* (maize) were retrieved from the National Center for Biotechnology Information (NCBI) [http://www.ncbi.nlm.nih.gov/] and Ensembl (http://ensembl.gramene.org/Zea_mays/Info/Index) and used as references. The obtained *SbPAL* sequences were aligned using the MUSCLE program (https://www.ebi.ac.uk/Tools/msa/muscle/)^37^. Key amino acids and important conserved sequences were identified by visual observation of *SbPAL* amino acid sequence alignment. A phylogenetic tree was constructed based on PAL amino acid sequences of rice, maize, wheat, Brachypodium, sorghum, and Arabidopsis, using the maximum likelihood method with poisson correction model (using 1000 boot strap values) in the MEGA7.0 program^38,39^. All DNA and RNA sequences used and analyzed during the current study are available in phytozome (https://phytozome.jgi.doe.gov/pz/portal.html) and additional data generated from this analysis are available in the supplementary materials/information with this paper.

### Identification of conserved motif, cis-element and feature analysis of sorghum PAL genes

To study structural diversity of *SbPALs*, conserved motifs were captured using the MEME program (http://meme-suite.org/tools/meme)^40^ with maximum number of motifs set at 20. To analyz*e Cis*-acting elements, the 2000 base pairs upstream from the ATG start codon of *SbPAL* genes were first obtained from Phytozome, followed by use of the PLANTRCARE database in the promoter regions^41^. TBtools (toolbox for biologists) v0.6741 was used to generate heat map to visualize expression of cis-acting element related to phytohormones, stress response and lignin biosynthesis in eight *SbPAL* promoters using the data presented in Supplementary Table S2. ExPASy bioinformatics program (https://www.expasy.org/) was used to predict Isoelectric point (PI) and molecular weight for each amino acid sequence. Sub-cellular localization of the protein was predicted using CELLO2GO software (http://cello.life.nctu.edu.tw/cello2go). Gene structure display server (GSDS) was used for analysis of exon and intron regions for PAL genes^42^.

### RNA extraction and gene expression analysis

Leaf samples were collected from uninfested (control) and infested plants of both genotypes at 6 hours (h), 12 h, 1 days (d), 2d, 3d, 6d and 9d post aphid infestation. The samples were immediately frozen in liquid nitrogen and stored in − 80 °C. Total RNA was extracted from flash-frozen stems and leaves of sorghum plants using TRIzol reagent (Invitrogen), followed by DNase treatment (Turbo DNA free kit, Thermofisher, Waltham, MA) for 30 min at 37 °C. cDNA was synthesized from 1.5 μg of total RNA using the GoScript reverse transcriptase kit (Promega, Madison, WI), following manufacture’s instructions. Primers were designed using the IDT DNA program (https://www.idtdna.com/PrimerQuest/Home/Index), which are listed in Supplementary Table S3. A sorghum β-tubulin gene (Sobic.002g350400) was used as the internal control^43^. Transcript levels were quantified by Quantitative real-time PCR (RT-qPCR) on a Bio-Rad icycler thermal cycler (Bio-Rad Laboratories, Inc., Hercules, CA, USA) using the iTaq™ universal SYBR^®^ green supermix (Bio-Rad Laboratories, Inc.). The total volume of qRT-PCR reaction was of 10 μl: 1 μl of diluted cDNA, 0.4 μl (10 μM) of reverse and forward primers each, 5 μl of SYBR green master mix and 3.2 μl of ddH2O. The following conditions were used: one cycle at 95 °C for 3 min, 40 cycles at 95 °C for 10 s and 55 °C for 30 s, followed by one cycle each of one min at 95 °C and 55 °C. The final melting curve was of 81 cycles at 55 °C for 30 s. Relative gene expression was calculated using the 2 − ΔΔCt method^44^ and the data were the average of three biological and two technical replicates.

### PAL activity assay

*PAL* activity assay was performed using a phenylalanine ammonia-lyase (PAL) microplate assay kit (Cohesion Biosciences) following the manufacturer’s instructions. Briefly, fresh 100 mg shoot tissue from uninfested (control) and SCA-infested sorghum plants, respectively, at different timepoints (6 h, 12 h, 1d, 2d, 3d, and 9d) were homogenized with 1 ml of assay buffer in a pre-chilled mortar and pestle. Homogenized samples were centrifuged at 8000× *g* for 15 min at 4 °C. The reaction mixture was prepared by adding 10 µl supernatant in 130 µl of reaction buffer and 50 µl of L-phenylalanine (substrate) in a 96-well plate, followed by 30 min incubation at 30° C. Stop solution (10 µl) was added to stop the reaction and PAL activity was determined by taking absorption spectra at 290 nm. The observance value from 2 technical and 5 biological replicates were used to calculate PAL activity using the following formula:

$${\text{PAL}}\left( {\text{U/g}} \right) = 66.7 \times \left( {{\text{OD}}_{{{\text{Sample}}}} - {\text{OD}}_{{{\text{Control}}}} } \right)/{\text{W}},$$

where one unit (U) is defined as the OD value change of 0.01 per minute; W, the weight of sample in g. The experiment was repeated two times.

### Exogenous SA treatment and its effect on SCA infestation in sorghum

Three leaf stage sorghum plants were treated with 50 mL of salicylic acid (SA) [100 ppm] or sterile water per plant by soil-drenching according to Pant et al.^24^, followed by SCA infestation after 24 h as described previously^45^. The second SA treatment was performed after 24 h of SCA infestation, followed by every three days for up to 9 dpi. The number of aphids on SA-treated and non-treated plants were recorded at 2, 3, 6, and 9dpi to determine the role of exogenous SA in aphid defense.

## Results

### Identification of PAL genes in sorghum and genomic analysis of their structures

To identify *SbPAL* gene family members, a homology search using *Brachypodium distachyon* PAL (*BdPAL*) amino acid sequences and key word search using “phenylalanine ammonia lyase” was performed against the *S. bicolor* genome database in phytozome (https://phytozome.jgi.doe.gov/pz/portal.html). As a result, 8 putative *PAL* genes were identified from sorghum and their characteristic features were studied (Table 1). Sobic.004G220300 was previously designated as *SbPAL1*^46^, and names of other genes are here designated as *SbPAL2*, Sobic.004G220400; *SbPAL3*, Sobic.004G220500; *SbPAL4*, Sobic.004G220600; *SbPAL5*, Sobic.004G220700; *SbPAL6*, Sobic.001G160500; *SbPAL7*, Sobic.006G148800; and *SbPAL8*, Sobic.006G148900. The length of *SbPAL* proteins varied from 703 (*SbPAL7*) to 718 (*SbPAL2*) amino acids, with calculated molecular weights ranging from 75.6 kDa (*SbPAL1*) to 76.82 (*SbPAL3* and *SbPAL4*). However, sorghum PAL proteins exhibit less variability in isoelectric points (PI) values, indicating that minor variation in ionic strength and/or pH range are required for their optimal activity. Most of the genes (five genes) are clustered on chromosome four, followed by two and one *PAL* on chromosome six and one, respectively. Subcellular localization of *SbPAL* proteins was predicted using CELLO2GO software and revealed that all sorghum PAL genes are localized in the cytoplasm (Table 1).

**Table 1:** The general information, structural features and properties of PAL genes of sorghum.

Name	Gene ID	Chromosome	Exon count	Strand	Location coordinates	Length (aa)	MW (KDa)	PI	Subcellular localization
SbPAL1	Sobic.004G220300	4	2	reverse	57051383..57055340	704	75.60	6.00	Cytoplasmic
SbPAL2	Sobic.004G220400	4	2	reverse	57064914..57069724	718	77.59	6.04	Cytoplasmic
SbPAL3	Sobic.004G220500	4	1	reverse	57075106..57077250	714	76.82	6.05	Cytoplasmic
SbPAL4	Sobic.004G220600	4	1	reverse	57083771..57086639	714	76.82	6.05	Cytoplasmic
SbPAL5	Sobic.004G220700	4	1	forward	57099422..57101566	714	76.80	6.04	Cytoplasmic
SbPAL6	Sobic.001G160500	1	2	reverse	13186392..13188966	709	76.24	5.60	Cytoplasmic
SbPAL7	Sobic.006G148800	6	2	reverse	51,039,816..51042905	703	75.69	6.25	Cytoplasmic
SbPAL8	Sobic.006G148900	6	2	reverse	51053477..51056309	714	76.60	5.80	Cytoplasmic

### Motifs and gene structural analysis of PAL genes in sorghum

**Figure 1:**
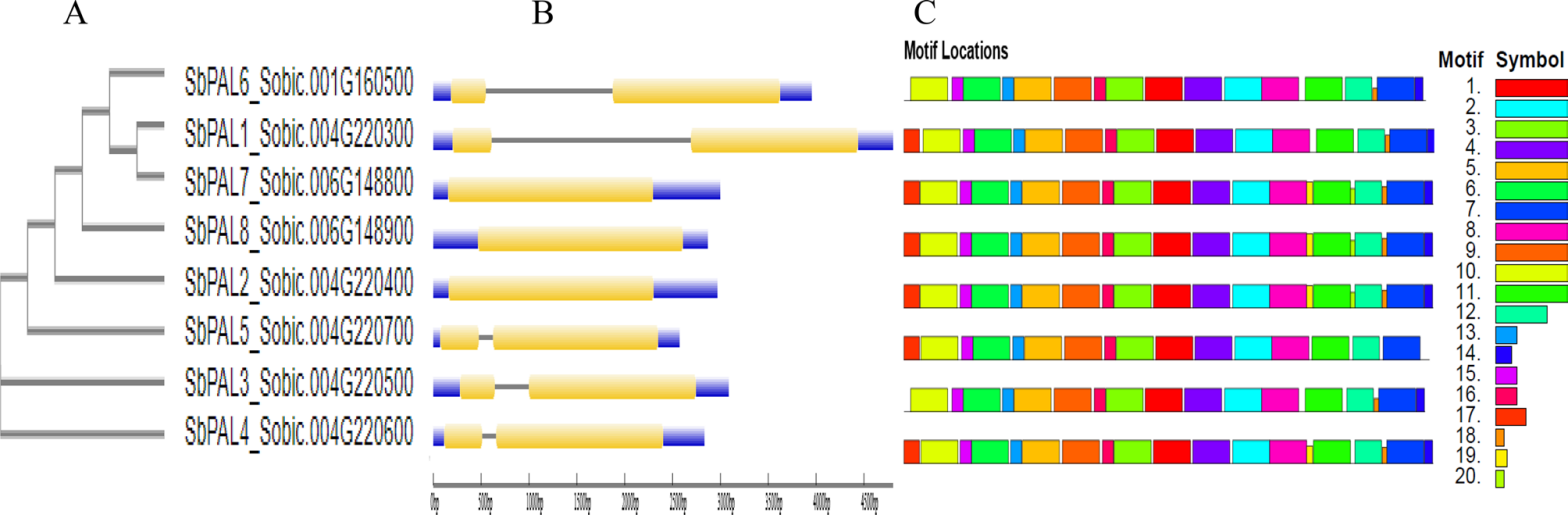
Phylogenetic relationship, gene structure and architecture of the conserved protein motifs of 8 PAL genes from sorghum. (**A**) The phylogenetic tree was constructed based on the full-length amino acid sequence. (**B**) Exon–intron structure of *SbPAL* genes in sorghum. Blue boxes represent untranslated 5′- and 3′-regions; gray line indicate intron and yellow boxes indicate exon. (**C**) The motif composition of *SbPAL* proteins. The motifs 1–20, are displayed in different color boxes. The sequence information of each motif is provided in Table 1.

To gain further understanding about sequence characteristics in *SbPAL*, we constructed a phylogenetic tree (Fig. 1A) and multiple sequence alignment (Supplementary Table 1) using the *SbPAL* amino acid sequences. The phylogenetic tree of *SbPAL* genes revealed that *SbPAL1* and *SbPAL7* are closely related and the other five members of this family (*SbPAL2*, *SbPAL3*, *SbPAL4*, *SbPAL5*, and *SbPAL8*) appear in different hierarchical positions on the tree. The *SbPAL6* is mostly related to *SbPAL1* and *SbPAL7* but far from the rest. Amino acid sequence alignment revealed that *SbPAL3* and *SbPAL4* shared identical amino acid sequence, which is also reflected in the phylogenetic tree. However, they differ in coding sequence as seen in exon–intron structure (Fig. 1B). Furthermore, amino acids sequence analysis also revealed that *SbPAL* differs in specific amino acid residue at position 123; histidine is present in *SbPAL1* and *SbPAL7* proteins, tyrosine is present in *SbPAL6* whereas phenylalanine is present in rest of the PAL proteins (Supplementary Table 1). In addition, *SbPAL6* has an asparagine at residue position 443 whereas lysine is present in the rest of the *SbPAL* proteins. A previous study stated that PALs with histidine in residue 123 are bifunctional and with phenylalanine in residue position 123 are monofunctional^46^.

To further study structural diversity of *SbPAL* genes, we predicted exon–intron and motif compositions in coding sequences using the Gene Structural Display Server (http://gsds.gao-lab.org/). The results showed that five genes possess two exons with varying lengths of intron sequences and rest of the three genes have single exon (Fig. 1B). Interestingly, all genes with single exon were clustered together in the phylogenetic tree (Fig. 1A). We identified 20 conserved motifs in *SbPAL* proteins using the MEME suite (http://meme-suite.org/). The result exhibits that 15 motifs were shared by all PAL proteins, however, rest of the five motifs were uniquely positioned in certain *SbPAL* (Fig. 1C). For example, motif 20 is uniquely positioned in *SbPAL3*, *SbPAL4*, and *SbPAL5* and motif 17 is absent in *SbPAL1* and *SbPAL7*. The sequence information of each motif is presented in Supplementary Fig. S1.

### Phylogenetic analysis of PAL in sorghum and other plants

**Figure 2:**
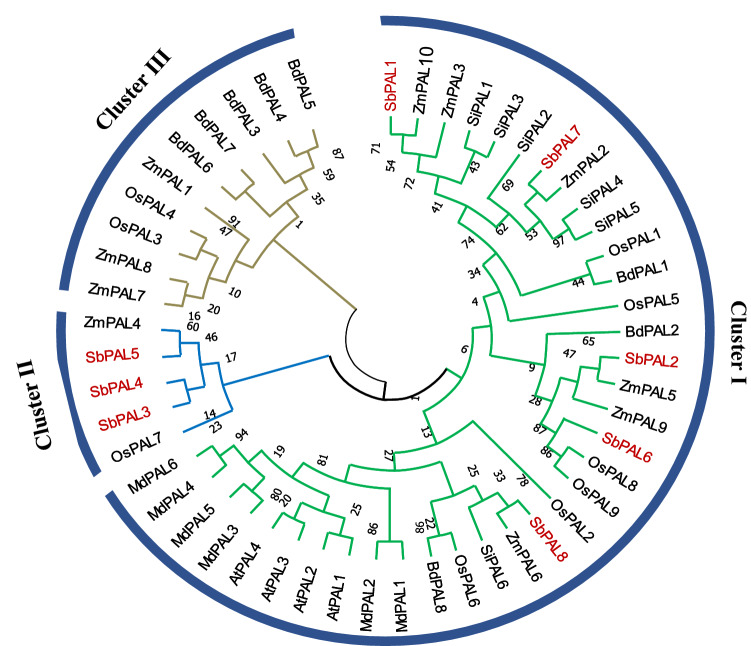
Phylogenetic analysis of the phenylalanine ammonia-lyase (PAL) genes in sorghum (*SbPAL*), rice (*OsPAL*), maize (*ZmPAL*), Brachypodium (*BdPAL*), barley (Horvu), and Arabidopsis (AtPAL).

To study the evolutionary relationship of sorghum PAL genes, a total of 51 amino acid sequences from monocotyledon crops (Brachypodium, rice, setaria, maize, sorghum) and the dicotyledon model plant Arabidopsis and apple were used to construct a phylogenetic tree using the maximum likelihood method (with 1000 bootstrap replicates) in MEGA5.0 software (Fig. 2). The phylogenetic tree categorizes all *PAL* genes into three clusters (Clusters I–III). Furthermore, *PAL* genes from each of monocot species are clearly distributed across the tree but as expected dicot plants (Arabidopsis and apple) formed a separate group. There are no dicot *PAL* genes in cluster II, which consists of the members from monocots only (maize, sorghum, and rice) and indicates that these *PAL* genes might have diverged after separation of monocotyledon and dicotyledon. As mentioned earlier, *SbPAL3*, *SbPAL4* and *SbPAL5* are highly similar, and aggregate together within cluster II. Interestingly, *SbPAL1* is in cluster I, and is indeed within a same sub-cluster as *OsPAL1* and *BdPAL1*, which are known to be involved in pathogen defense in host plant, suggesting a similar defense function of *SbPAL1* in sorghum.

### Comparative expression analysis of *PAL* genes during sugarcane aphid infestation

To get insight into the role of *SbPAL* genes during SCA infestation in sorghum, we analyzed their gene expression in shoot tissues from resistant and susceptible sorghum lines using qRT-PCR. We collected shoot tissues from early and late stages of SCA infestation. The samples collected at 6 h, 12 h, 24 h and 48 h were categorized as early stage and those collected at 3d, 6d and 9d after infestation were grouped as late stage. Since *SbPAL3* and *SbPAL4* are identical in amino acid sequence, we have only selected *SbPAL3* for gene expression analysis. The transcript levels of *SbPAL6* and *SbPAL8* were undetectable or very low in both control and SCA infested samples, hence, were excluded from gene expression analysis. For comparative purposes, the expression of each gene under control conditions was normalized to 1.

**Figure 3:**
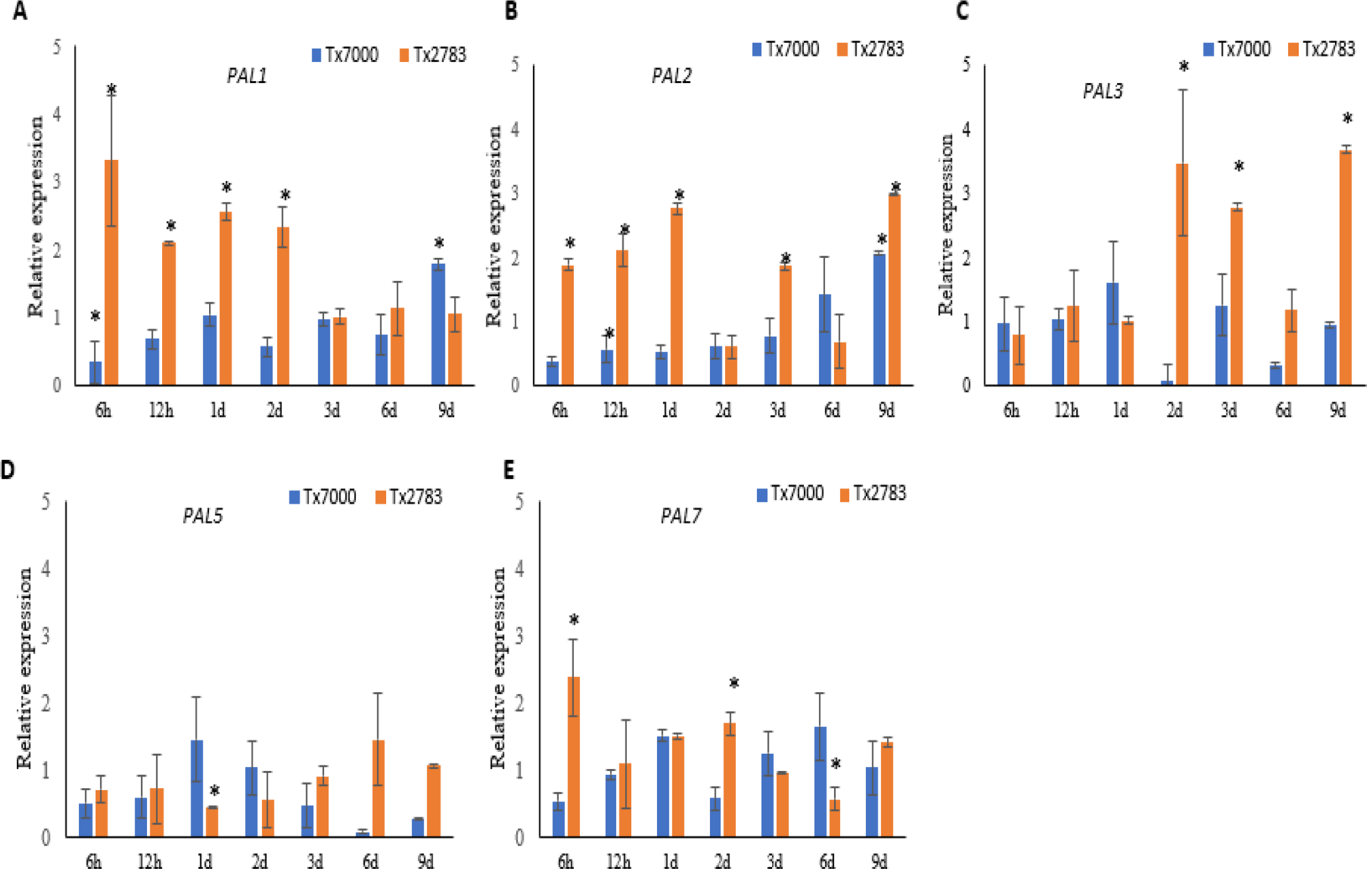
Gene expression analysis of five *SbPAL* genes (*PAL1*, *PAL2, PAL3, PAL15,* and *PAL7*) in controls and sugarcane aphid (SCA) infested shoot tissues from two sorghum lines (Tx7000, Tx2783). qRT-PCR was used to determine the relative expression of each *SbPAL* gene, and the relative expression was estimated using the 2^−ΔΔCt^ method. Samples were collected at 6 hours post infestation (h), 12 h, 1 day post infestation (d) 2 d, 3 d, 6 d, and 9 d. Gene expression in control (none-infested) plants of each line is normalized to 1. Error bars represent standard error among replicates (n = 3) and the asterisks represent statistically significant changes between the controls and SCA infested samples as determined using Student’s t-test (*p* ≤ 0.05), while the bars without asterisk are non-significant (*p* > 0.05).

In SCA infested samples, *SbPAL1*, *SbPAL2,* and *SbPAL7* showed a similar trend, and their expressions were significantly induced compared to the uninfested control in the resistant sorghum line at most of the time points of early stage of infestation (Fig. 3A, B). Whereas their expressions in the susceptible line were not significantly increased in the early stage of SCA infestation (Fig. 3). However, their expression on average slowly dropped from their peaks at early stage of infestation. At 9 days post infestation (dpi), the expression of *SbPAL2* and *SbPAL3* genes were significantly elevated again in compared to un-infested plants. We didn’t observe any significant induction of *SbPAL5* expression in either resistant or susceptible lines at most time points except in the resistant line at 6 dpi and 9 dpi following SCA infestation (Fig. 3D, E). Interestingly, *SbPAL1* and *SbPAL2* exhibited significant upregulated at 9 dpi in the susceptible line. *SbPAL3* showed the highest level of expression at 48 h post infestation (hpi) and consistently showed elevated expression up to 9 dpi in the resistant line. Together, the qRT-PCR analyses revealed that *SbPAL* genes have differential expression in resistant and susceptible lines, and they are most likely involved in host defense against SCA in a time dependent manner.

### Assessment of PAL activity in resistant and susceptible plants

**Figure 4:**
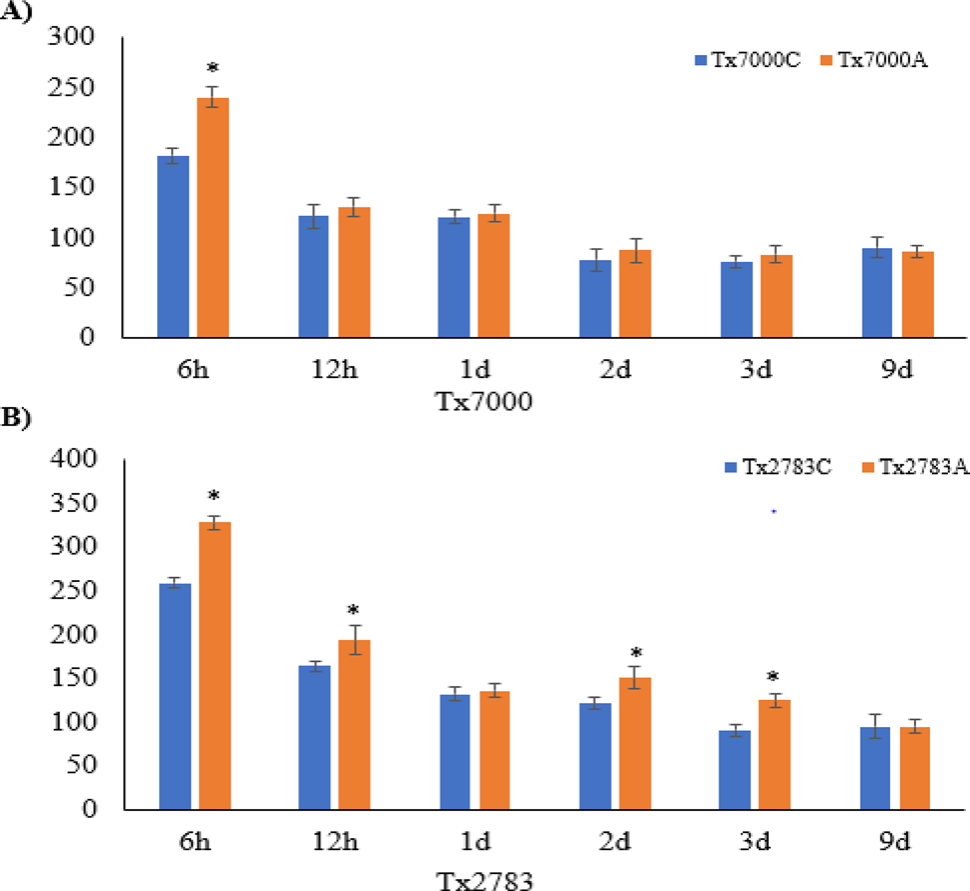
Comparison of the PAL activities between sugarcane aphid (SCA) infested sorghum plants and in the controls at various time points after exposure to SCA, and also the PAL activities shown in susceptible genotype Tx7000 (**A**) and resistant genotype Tx2783 (**B**), respectively. Error bars represent standard error among replicates. The asterisks represent statistically significant changes between the controls and SCA infested plants as determined using Student’s t-test (*p* ≤ 0.05).

To determine whether the changes in *PAL* expression mirrored in PAL activity, we performed PAL activity assays to detect the formation of trans-cinnamate from L-phenylalanine in SCA susceptible and resistant sorghum lines after 6 h, 12 h, 1d, 2d, 3d and 9d post-infestation with SCA. The results revealed that on average, the SbPAL activities were higher in the resistant line compared to the susceptible line (Fig. 4). Moreover, SCA infestation triggered SbPAL activities in both sorghum lines at all time points.

### Analysis of *cis*-acting elements in *SbPAL* gene promoter region

**Figure 5:**
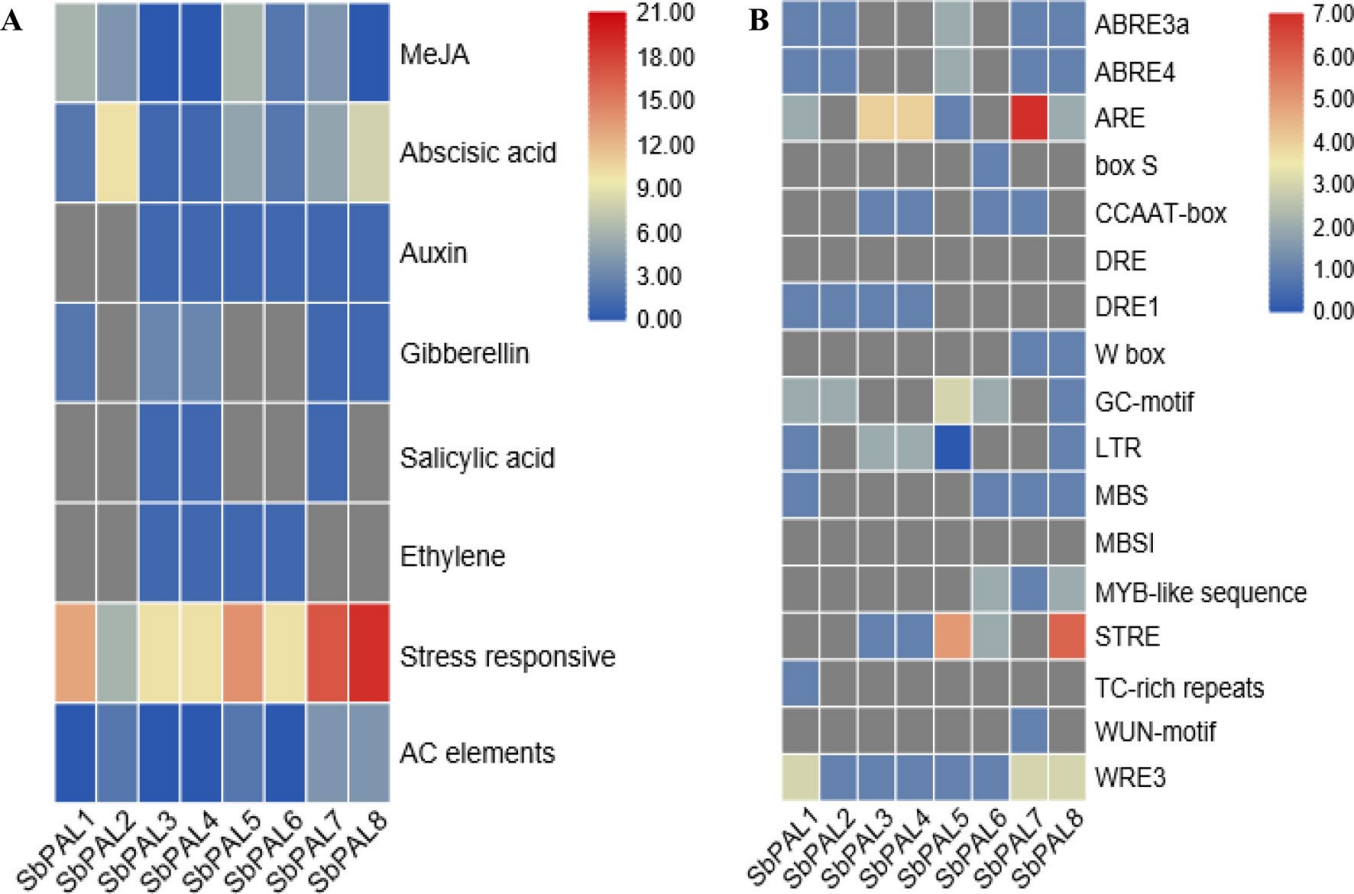
Heat map view of the sum of putative cis-acting elements related to phytohormones, stress response and lignin biosynthesis (**A**), and the specific number of stress related cis-acting elements (**B**) in eight sorghum PAL promoters. Color bars represent number of putative cis-acting elements present in each PAL gene. This heat map was created by the authors using TBtools (toolbox for biologists) v0.6741 from the data presented in Supplementary Table S2.

To better understand the transcriptional regulation of *SbPAL*s, we analyzed the promoter region by scanning 2000 bp sequences of the initiation codons of *SbPAL* genes using the PlantCARE tool^41^ (Fig. 5A, B). The PlantCARE tool has predicted dozens of cis-acting elements in *SbPAL* genes (Supplementary Table S2). Among them, several cis-acting elements belong to phytohormone response and lignin biosynthesis (AC-elements). The *cis*-acting elements analysis showed that *SbPAL* genes are mainly regulated by the following phytohormones: methyl jasmonate (MeJA), abscisic acid (ABA), auxin (IAA), gibberellin (GA), and salicylic acid (SA). The sum of the *cis*-acting elements related to these hormones are presented in Fig. 5A. We predicted several stress-related *cis*-acting elements that could regulate the *SbPAL* expression in response to biotic and abiotic stress. The common *cis*-acting elements related to stress are anaerobic, defense, drought, low temperature, and wound, among which anaerobic-related (ARE) cis-acting elements were the most common, followed by those defense-related elements (STRE and TC-rich repeats) and the binding set of the MYB transcription factor (MYB-like sequence) (Fig. 5B). AC-elements which regulated the biosynthesis of lignin, was detected from promoter regions of *SbPAL.* The AC-elements were found in abundant number in *SbPAL1, SbPAL3, SbPAL4,* and *SbPAL6,* indicating their possible role in lignin synthesis in sorghum. The list of all *cis*-elements identified in promoter region of *PAL* genes in sorghum are presented in Supplementary Table S2.

### Effect of exogenous application of SA on host plants during SCA infestations

**Figure 6:**
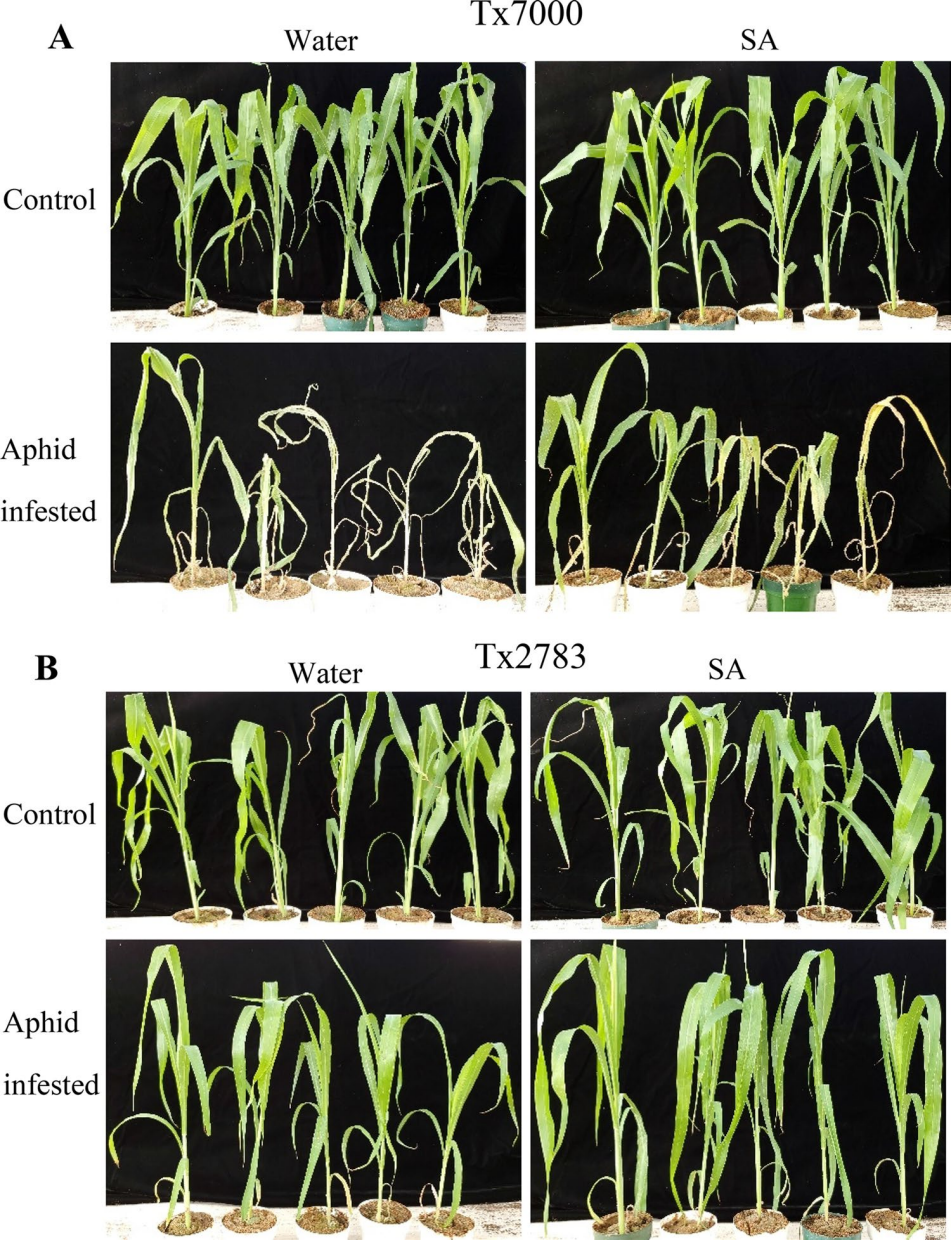
Effect of exogenous application of salicylic acid (SA) on reducing aphid damage in susceptible line Tx7000. Representative Tx7000 (**A**) and Tx2783 (**B**) plants at 12 days post infestation (dpi) of sugarcane aphids.

**Figure 7:**
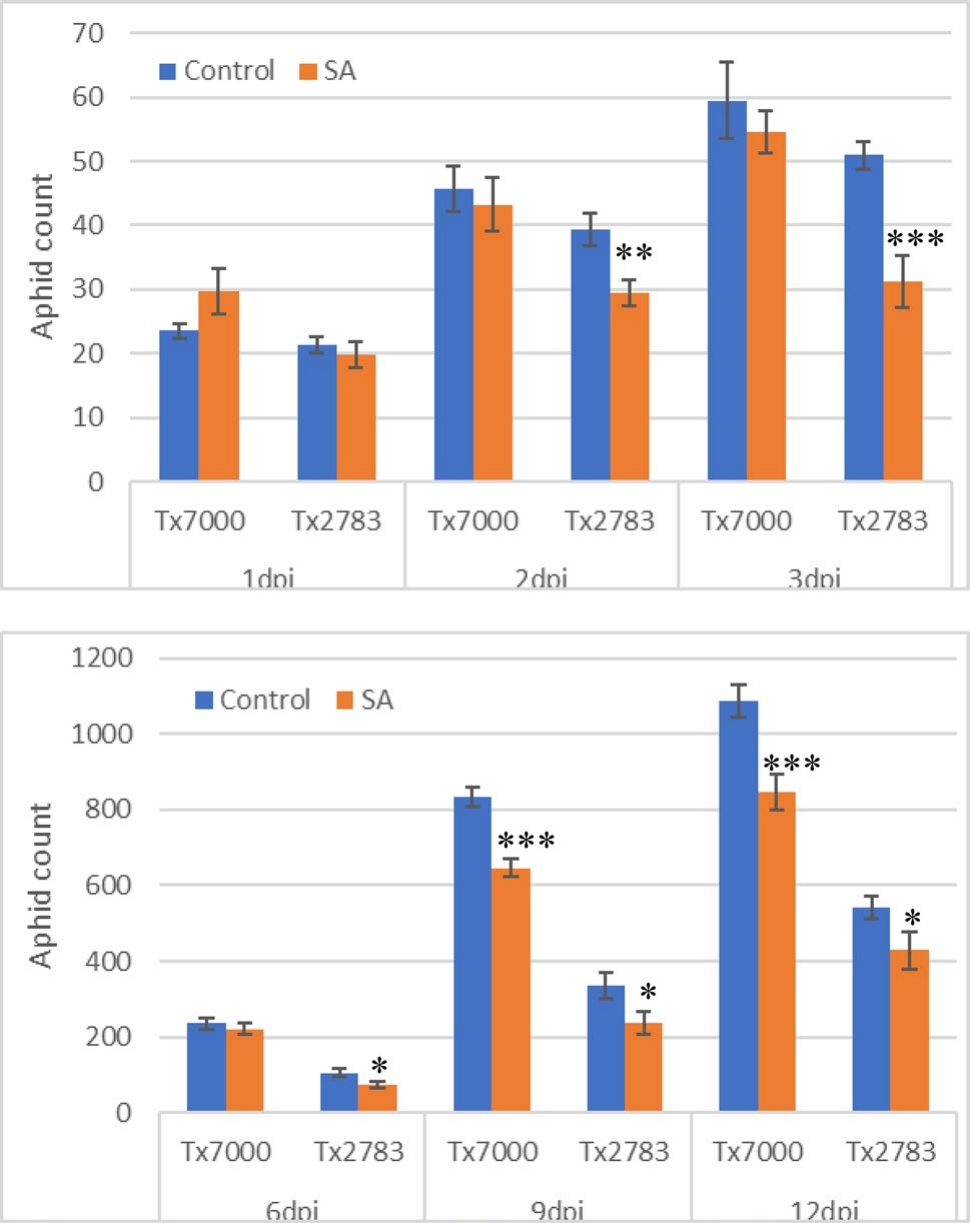
Aphid count in control and SA treated sorghum lines at 1-, 2-, 3-, 6-, 9- and 12-days post infestation of sugarcane aphid (SCA). Error bars represent standard error among replicates. The asterisks represent statistically significant changes between control and SA treated samples as determined using Student’s t-test (*, *p* ≤ 0.05; **, *p* ≤ 0.01; ***, *p* ≤ 0.001).

*PAL* is an important enzyme of SA biosynthesis pathway. We have demonstrated via qRT-PCR that *PAL* expression is induced after SCA infestation in sorghum. It is well established that *PAL* contributes to activate SA induction in response to biotic stresses^14,20,21,47,48^. We were interested to evaluate if exogenous application of SA could restore susceptibility phenotype of Tx7000 line. For this, we treated plants with SA and studied the plant phenotype, and survival and fecundity of SCA. We found that treatment with SA has a dramatic effect on infestation phenotype, and survival and reproduction in sorghum. Exogenous application of SA partially reduced the SCA susceptible phenotype (e.g., chlorosis, necrosis), which is more pronounced in susceptible lines compared to resistant lines (Fig. 6). In addition, plants treated with SA survived longer compared to untreated plants during the infestation testing when infested with SCA. For instance, the Tx7000 plant usually died two weeks after SCA infestation, whereas after SA treatment, it was able to survive up to three weeks (data not shown). We also counted the number of aphids in the SA-treated and untreated sorghum lines. We found that SA treatment significantly decreased the number of aphids in both resistant and susceptible lines. These differences were more evident in susceptible line, especially in late-stage infestations (9 and 12 dpi) (Fig. 7). Taken together, these results suggest that an induced level of SA plays an important role in resistance to SCA infestation by moderating symptom severity and limiting SCA fecundity.

## Discussion

PALs are widely studied plant enzymes in response to biotic and abiotic stresses in plants^1,4,13,20,21^. PALs have also been reported recently, showing their response to aphids in crop plants^11,23,47,49^. In this study, we focused on molecular characterization of the *PAL* gene family and their functional role in SCA resistance in sorghum using bioinformatics, phenotypic and molecular approaches, and here we reported that SbPAL is involved in host resistance to SCA infestation in sorghum.

The PAL enzyme is encoded by a multigene family in plants. The numbers of PAL genes vary in different species, for example, Arabidopsis and tobacco has four PALs^20,50,51^, rice has nine^52^, and Brachypodium has eight^21^. In this study we identified eight genes in sorghum localized in chromosome 1, 4 and 6, with most of them clustered in chromosome 4 (Table 1). Compared to the number of *PAL* genes in dicot model plants (Arabidopsis and tobacco), the number of *PAL*s in grass family are higher. The increase in number of genes is considered a natural process of frequent duplication and long evolution. Jun et al.^46^ found that amino acid residue at position 123 determines specificity to it substrates in *SbPAL* proteins. Through sequence alignment, we found that at position 123 histidine is present in *SbPAL1* and *SbPAL7*, tyrosine is present in *SbPAL6* and phenylalanine is present in the rest of the PAL proteins. PALs with histidine at residue 123 are bifunctional and displayed both PAL and TAL activities, whereas with phenylalanine residue, they are monofunctional and only have PAL activity^46,53^. One bifunctional PAL protein has been identified from Brachypodium (*BdPAL1*) and maize (*ZmPAL1*)^8,53^. The variation in number of bifunctional PAL enzymes in closely related grass families suggest an inherent variation among grass species to regulate metabolic flux to respond specific abiotic and biotic stresses^46^. To study whether *SbPAL* genes are conserved in gene sequences, we performed gene structure and motif analysis. *SbPAL2*, *SbPAL7* and *SbPAL8* are intronless and are positioned together in the phylogenetic tree (Fig. 1A). The remaining five *SbPALs * have single introns and are diverged in the phylogenetic tree based on their varying lengths and sequences of intron. The intron–exon pattern in a gene family reflects the evolutionary history of the gene family^54,55^. In this study, we identified 20 distinct conserved motifs in *SbPAL* (Fig. 1C), most of which are conserved among the *SbPAL* family. The results also revealed a few unique motifs whose compositions contribute to the functional diversity of *SbPAL* genes. The high level of motif conservation in *SbPAL* suggests conserved biological functions of PAL family members in sorghum.

The biological function of the *SbPAL* genes were analyzed in both resistant and susceptible sorghum plants in response to SCA infection at multiple time points. We found different expression patterns among the *SbPAL* genes, for instances, *SbPAL6* and *SbPAL8* showed low expression in sorghum shoots and were excluded in this study. Low expression of these two PAL genes were further supported by expression data in the phytozome, which suggested that *SbPAL8* is only expressed in roots and *SbPAL6* expressed in low levels in the internode region. Expression of remaining genes were significantly induced except *SbPAL5* in a time-dependent manner upon exposure to SCA in the resistant line (Fig. 3). However, variance of *SbPAL* expression level was not significant in the susceptible line. Elevated expression of the *SbPAL* genes in the resistant sorghum line suggests a strong correlation between upregulation of the *SbPAL* genes and SCA resistance. Previous reports have shown differential expression of genes belonging to the phenylpropanoid biosynthetic pathway in the susceptible and resistant lines^21,48,56^. Tetreault et al.^56^ reported down-regulation of *SbPAL* genes in the susceptible line (BCK60) at 10- and 15-days post SCA infestation. But Grover et al.^49^ showed a suppression of phenylpropanoid proteins on day 1 after SCA infestation, while upregulated on day 7 in a different sorghum line. It is a little inconsistency but is understandable that differential expression of the PAL genes was not consistent among those reports because they were not the same gene though they all belong to the PAL gene family. Apparently, individual genes of the PAL family exhibit very different patterns in response to SCA attack and changes in expression levels at different time points as shown in Fig. 3. This suggests that each member of the PAL gene family plays a different role in the host plant and acts at certain times during the interaction between the host plant and the aphids.

In choice assays, authors showed that SCA has strong preference for the susceptible genotype (BCK60) and have a higher fecundity rate when compared to Tx2783. The authors also found that PALs are localized in cytoplasm and Tx2783 that provide phloem-based resistance, which further confirm other reports about the existence of aphid-resistance factors in phloem^22,57,58^. Pant and Huang^24^ also reported higher SCA fecundity in the susceptible line (Tx7000) when compared to the resistance line (Tx2783) which could co-relate to low accumulation of phenylpronoid accumulation along with other resistance factors such as phloem protein coagulation, callose and lectin deposition, and other secondary metabolites^59–62^. Induction of *SbPAL* genes and PAL enzyme activity in the resistance line during SCA infestation suggests that the accumulation of phenylpropanoid products in phloem during infestation could have led to the deterrence of SCA.

A strong induction of *SbPAL1* at an early stage of infestation suggests its primary defense role in SCA infestation in sorghum. Our in-silico analysis and phylogenetic analysis showed that *SbPAL1* and *SbPAL7*, the bifunctional proteins, are closely associated with Brachypodium bifunctional PAL protein (*BdPAL1*) and rice PAL (*OsPAL1*). The role of *BdPAL1* in pathogen defense was well documented against *Magnaporthe* sp., *Fusarium cuimorum* and *Panicum mosaic virus*^21,48^. Overexpression of *OsPAL1* in susceptible variety enhanced resistance to *Magnaporthe oryzae*^63^. Potential cis-elements associated with stress response like salicylic acid and wounding, and MYB-like sequences, were predicted from promoter regions of *SbPAL1*, which could drive an overexpression during SCA infestation. For example: An R2R3 MYB transcription factor confers brown plant hopper resistance by inducing expression of the majority of phenylalanine ammonia-lyase genes in rice^64^. Pandey et al.^65^ demonstrated that insecticidal proteins were significantly induced under a wound-inducible promoter from rose (RbPCD1pro) in the early stage of *Helicoverpa armigera* and *Myzus persicae* infestation, and transgenic plants showed strong resistance against insects. These findings imply *SbPAL1* expression was induced early upon SCA infestation in sorghum. However, further studies are needed to understand the functional role of these promoters in SCA resistance in sorghum. The heat map indicated that *SbPAL1*, *SbPAL5*, *SbPAL7* and *SbPAL8* have a higher number of stress responsive *cis*-elements. Interestingly, *SbPAL7* has a greater number of stress responsive and anaerobic stress (ARE)- and wound (WEE3)- responsive and W-box elements, which is consistent with its relatively higher expression in shoots upon SCA infestation (Fig. 3). W-box is a binding site for the WRKY transcription factor and commonly present in the promoter region of *PR* genes and is induced by SA. A prior study^66^ suggested that the W-box plays an important role in SAR and has been identified in promoter regions of PAL genes in cucumber and rice^67^ and proposed for their role in SAR and SA-induced defense. Moreover, AC elements are also found from the promoter regions of *SbPAL*. AC elements are abundant in lignin biosynthesis genes and induce lignin monomer biosynthesis by binding to the MYB transcription factor^68–70^. Therefore, higher number of AC elements in promoters of *SbPAL1*, *SbPAL3*, *SbPAL4* and *SbPAL6* suggest that these genes may be involved in the lignin biosynthesis in sorghum.

To further understand the role of *SbPAL* in SCA resistance in sorghum, a PAL activity assay was performed which affirmed that PAL activity was higher in the resistant line compared to susceptible plants. These findings are aligned with a recent report of proteome analysis in resistant sorghum genotype (SC265), which showed induced expression of PAL protein after 7 days post SCA infestation^49^. These results suggest that PAL enzymes play a crucial role conferring resistance to SCA infestation in sorghum. An elevated level of *PAL* expression and activity were also commonly observed in other plants during their reactions to pests/pathogens. For example, *PAL* expression were upregulated in beans, sorghum, Brachypodium, and rice in response to fungal infection^11,21,47^, in Brachypodium and maize to virus infection^23,48^, in *Lotus japonicus* to *Rhizobium* symbiosis^71^, in pepper and beans to bacterial pathogens^3,14^ and in rice to broad spectrum disease resistance^72^. These studies suggested that the defense role of PALs in plants may be due to accumulation of SA and phenylpropanol intermediates. Furthermore, we investigated whether exogenous application of SA on plants would affect the SCA infestation in susceptible sorghum line. From this study, we demonstrated that the susceptible plant treated with SA alleviated the susceptibility phenotype and significantly reduced the number aphids on the plant compared to SA-untreated plants. Furthermore, SA-treated susceptible plants exhibited delays in the development of the damage symptoms such as chlorosis, necrosis, and subsequent plant death by 5–7 days (Fig. 7). Similar observations were also reported in recent studies, which demonstrated a higher accumulation of SA level in SCA resistant genotype at multiple timepoints^49,73,74^. Furthermore, the recent research results showed upregulation of SA production in sorghum plants following SCA infestation relatively to uninfested plants in both resistant and susceptible genotypes and the levels of SA increased continuously starting from 1-, 3-, through 6-dpi^75^. In the report, the aphid-induced SA production in sorghum plants was also supported by the upregulated expression of *SbPAL* genes. SA and JA phytohormones behave antagonistically, and defense role of SA in SCA infestation is further supported by a recent study in JA in sorghum^73^. The authors found that JA-deficient sorghum plant reduced aphid feeding and population while exogenous supply of JA attenuated the resistance phenotype and enhanced aphid feeding and population. These observations further support the positive correlation in SA level and aphid resistance in sorghum. Interestingly, exogenous application SA failed to fully restore resistance phenotype to the level of resistant genotype, which entails the existence of multiple resistance factors in phloem in addition to SA.

Taken together, these results suggest that *SbPAL* confers resistance in sorghum to SCA via SA-dependent defense signaling, which is consistent with the published results^75^. In our results from *PAL* expression, PAL activity and SA treatment experiments suggest that certain threshold levels in PAL protein and SA were required to confer the resistance to aphid. Susceptible lines failed to meet the minimum threshold level; thus, the plant became susceptible to SCA. However, more studies are required to demonstrate a direct connection between PAL induction and the salicylic acid-mediated defensive response of the plant. Prior studies demonstrated via PAL-silencing experiments in Arabidposis, Brachypodium, maize, and pepper, that PAL plays an important role in SA-dependent signaling of the defense response to plant-pest and pathogen infections^13,14,17,18,21,23^. Thus, these results confirmed that the SbPALs play an important role in SCA resistance in sorghum via SA-dependent defense signaling.

## Conclusion

The present study provides the first analysis for motifs, promoters, gene structure, and phylogenetic classification of the *PAL* gene family in sorghum. Furthermore, our comparative gene expression studies of sorghum in response to SCA between susceptible and resistant lines demonstrated that members of the *SbPAL* family genes expressed differently during SCA infestation. We showed that SCA infestation in sorghum triggered the induction of *SbPAL* activation in both susceptible and resistant genotypes. However, strong expression was observed in the resistant line, suggesting that a certain threshold of PAL enzyme activity is required to confer resistance during SCA infestation. A higher PAL enzyme activity in the resistant line further supports that PAL contributes to the host resistance during SCA infestation in sorghum. Exogenous application of SA alleviates the susceptibility phenotype and suppresses the growth and reproduction of SCA in the susceptible line, suggesting an important role of PAL in SCA tolerance via SA-mediated defense signaling. Overall, the findings of our study broaden the understanding of the defense role of *PAL* in sorghum and offer an opportunity to further dissect its role in host resistance to other pests and diseases.

## Supplementary Information

Supplementary Information.

## Data Availability

All data generated during this study are included either in main text or as supplementary data; otherwise, additional data can be requested from the corresponding author.
