# Supplementary material for: Genome-wide studies of PAL genes in sorghum and their responses to aphid infestation
Source: Sci Rep. 2022 Dec 29;12:22537. doi: 10.1038/s41598-022-25214-1 (PMC9800386; doi:10.1038/s41598-022-25214-1)
Supplement: Supplementary file 1 — Supplementary Information. [file 41598_2022_25214_MOESM1_ESM.pdf]

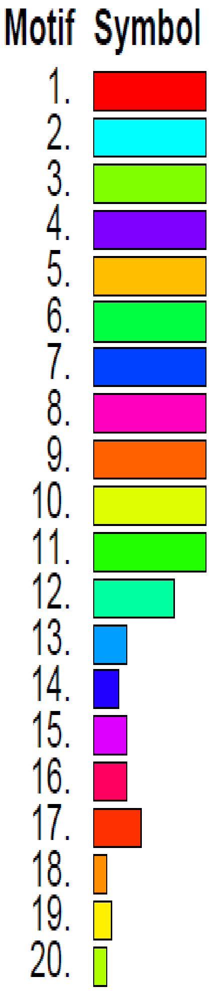

Motif Symbol Motif Consensus

1. LDPLMKPKQDRYALRTSPQWLGPQIEVIRAATKSIEREINSVNDNPLIDV
2. RNPSLDYGFKGAEIAMASYCSELQFLGNPVTNHVQSAEQHNQDVNSLGLI
3. SAVFCEVMNGKPEYTDHLTHKLKHHPGQIEAAAIMEHILEGSSYMKLAKK
4. KALHGGNFQGTPIGVSMDNTRLAIAAIGKLMFAQFSELVNDYYNNGLPSN
5. RINTLLQGYSGIRFEILEAIAKLLNANVTPCLPLRGTITASGDLVPLSYI
6. SSDWVMBSMMNGTDSYGVTTGFGATSHRRTKEGGALQRELIRFLNAGAFG
7. CRSYPLYRFVRZELGAVYLTGEKTRSPGEELNKVLVAINQGKHIDPLLEC
8. SSRKTAEAIDILKLMSSTFLIALCQAVDLRHJEENVKSAVKSCVMTVAKK
9. TGRENSVAVAPDGTKVNAAEAFKIAGIQGGFFELQPKEGLAMVNGTAVGS
10. ADPLNWGKAAEELTGSHLDEVKRMVAEYRQPLVKIEGASLRIAQVAAVAA
11. LHVARFCEKDLLTAIDREAVFAYADDPCSANYPLMKKLRAVLVEHALANG
12. ETSVFAKVAZFEEELRAALPKEVEAARAAVENGTAP
13. DGHVLPAEATRAAML
14. KEWNGEPLPIC
15. ARVELDESARGRVKA
16. TVLFEANVLAILAEV
17. MECENGRVAATNGDGLCMATP
18. PNRITE
19. TLSTNSTG
20. AAEFDA

Supplementary Figure 1. The sequence information of each motif presented in **Fig. 1C.**

Supplementary Table 1. Omega clustalw alignment of full-length amino acid sequence from eight PAL genes in sorghum. Green color represents amino acid residue at position 123 and Gray color represents amino acid residue at position 443.

```
Sobic.001G160500      MECETGLVDRPRRNGSA---GPHVVDDDPLKWGKAAEDLTGSHLDDVRRMVEEYRMPLVK    57
Sobic.006G148900      MECENGHAAGASGNGVCLAT---PRAADPLNWGKAAEDLTGSHLEAVKRMVEEYRRPLVK    57
Sobic.004G220400      MECETGLVRSLNGDGLCMSAQAAPRGADPLNWGKAAEDLSGSHLEEVKRMVAEFRDPVVK    60
Sobic.004G220500      MACENGRVAATNGDGLCMATP----RADPLNWGKAAEELMGSHLDEVKRMVAEYRQPLVK    56
Sobic.004G220600      MACENGRVAATNGDGLCMATP----RADPLNWGKAAEELMGSHLDEVKRMVAEYRQPLVK    56
Sobic.004G220700      MACDNGRVAATNGDGLCMATP----RADPLNWGKAAEELMGSHLDEVKRMVAEYRQPLVK    56
Sobic.004G220300      -----------------MAGNGAIVESDPLNWGAAAAELSGSHLDEVKRMVAQARQPVVK    43
Sobic.006G148800      -----------------MASNTAILESDPLSWGKAAAELTGSHLDEVKRMVAQFRDPVVK    43
                                                 ***.** ** :* ****: *:*** : * *:**

Sobic.001G160500      IDGAMLSVAKVAAVAGE-ATRRVQVVLDESARPRLEASREWVFDSTMNGTDTYGVTTGFG    116
Sobic.006G148900      IEGASLTVAQVAAVA-AA--GEARVELDESARGRVKASSDWVMNSMMNGTDSYGVTTGFG    114
Sobic.004G220400      IQGASLSIAQVAAVAAGA--GEARVELDESARERVKASSDWVMSSMMNGTDSYGVTTGFG    118
Sobic.004G220500      IEGASLRIAQVAAVAAGA--GEARVELDESARGRVKASSDWVMNSMMNGTDSYGVTTGFG    114
Sobic.004G220600      IEGASLRIAQVAAVAAGA--GEARVELDESARGRVKASSDWVMNSMMNGTDSYGVTTGFG    114
Sobic.004G220700      IEGASLRIAQVAAVAAGA--GEARVELDESARGRVKASSDWVMNSMMNGTDSYGVTTGFG    114
Sobic.004G220300      IEGSTLRVGQVAAVASAKDASGVAVELDEEARPRVKASSEWILDCIAHGGDIYGVTTGFG    103
Sobic.006G148800      IEGSTLRVGQVAAVAAAKDASGVAVELDEEARPRVKASSEWILDCIAHGGDIYGVTTGFG    103
                      *:*: * :.:*****       . * ***.** *::** :*::..  :* * ********

Sobic.001G160500      GASHRRTKEFRALQTELIRYLNAGVFGASSSDGYTLPAEVTRAAMLVRINTLTQGYSGIR    176
Sobic.006G148900      ATSHRRTKEGGALQRELIRFLNAGAFGTG-DDGHVLPAAATRAAMLVRINTLLQGYSGIR    173
Sobic.004G220400      ATSHRRTKEGGALQRELIRFLNAGAFGTG-ADGHVLPAEATRAAMLVRINTLLQGYSGIR    177
Sobic.004G220500      ATSHRRTKEGGALQRELIRFLNAGAFGTG-TDGHVLPAEATRAAMLVRINTLLQGYSGIR    173
Sobic.004G220600      ATSHRRTKEGGALQRELIRFLNAGAFGTG-TDGHVLPAEATRAAMLVRINTLLQGYSGIR    173
Sobic.004G220700      ATSHRRTKEGGALQRELIRFLNAGAFGTG-TDGHVLPAEATRAAMLVRINTLLQGYSGIR    173
Sobic.004G220300      GTSHRRTKDGPALQVELLRHLNAGIFGTG-SDGHTLPSEVVRAAMLVRINTLLQGYSGIR    162
Sobic.006G148800      GTSHRRTKDGPALQVELLRHLNAGIFGNG-SDGHTLPSEVSRAAMLVRINTLLQGYSGIR    162
                      .:******:  *** **:*.**** ** .  **:.**: . *********** *******

Sobic.001G160500      FEILEAIAKLLNANVTPCLPLRGSITASGDLVPLSYIAGLITGRGNSAAVAPDGTKVGAA    236
Sobic.006G148900      FEILETIAALLNANVTPCLPLRGTITASGDLVPLSYIAGLVTGRPNSTAVAPDGRKVGAA    233
Sobic.004G220400      FEILEAIAKLLNANVTPCLPLRGTITASGDLVPLSYIAGLITGRQNSVAVAPDGRKVDAA    237
Sobic.004G220500      FEILEAIVKLLNANVTPCLPLRGTVTASGDLVPLSYIAGLVTGRENSVAVAPDGTKVNAA    233
Sobic.004G220600      FEILEAIVKLLNANVTPCLPLRGTVTASGDLVPLSYIAGLVTGRENSVAVAPDGTKVNAA    233
Sobic.004G220700      FEILEAIVKLLNANVTPCLPLRGTVTASGDLVPLSYIAGLVTGRENSVAVAPDGTKVNAA    233
Sobic.004G220300      FEILEAITKLLNTGVSPCLPLRGTITASGDLVPLSYIAGLITGRPNAQATTVDGRKVDAA    222
Sobic.006G148800      FEILEAITKLLNTGVSPCLPLRGTITASGDLVPLSYIAGLITGRPNAQAVTVDGRKVDAA    222
                      *****:*. ***:.*:*******::***************:*** *: *.: ** **.**

Sobic.001G160500      EAFRIAGIDHGFFALEPKEGLAIVNGTAVGSGLAAIVLFEANVLAVLAEVLSAVYCEVMA    296
Sobic.006G148900      EAFKIAGIQHGFFELQPKEGLAMVNGTAVGSGLASMVLFEANVLAVLAEVMSAVFCEVMN    293
Sobic.004G220400      EAFKIAGIEHGFFELQPKEGLAMVNGTAVGSGLASTVLFEANVLAIMAEVISAVFCEVMT    297
Sobic.004G220500      EAFKIAGIQGGFFELQPKEGLAMVNGTAVGSGLASTVLFEANVLAILAEVLSAVFCEVMN    293
Sobic.004G220600      EAFKIAGIQGGFFELQPKEGLAMVNGTAVGSGLASTVLFEANVLAILAEVLSAVFCEVMN    293
Sobic.004G220700      EAFKIAGIQGGFFELQPKEGLAMVNGTAVGSGLASTVLFEANVLAILAEVLSAVFCEVMN    293
Sobic.004G220300      EAFKIAGIEGGFFKLNPKEGLAIVNGTSVGSALAATVMYDANVLAVLSEVLSAIFCEVMN    282
Sobic.006G148800      EAFKVAGIQGGFFKLNPKEGLAIVNGTSVGSALAAMVCFDANVLAVLSSVLSAVFCEVMN    282
                      ***::***: *** *:******:****:***.**: * ::*****:::.*:**::****

Sobic.001G160500      GNPEYTDHLIHALKHHPGQIEAAAIMEHVLEGSSYMKLAREQAELDPLTKPKQDRYAIRT    356
Sobic.006G148900      GKPEYTDHLTHKLKHHPGQIESAAIMEHILDGSSYMMLAKKMGELDPLMKPKQDRYALRT    353
Sobic.004G220400      GKPEFTDHLTHKLKHHPGQIEAAAIMEHILEGSSYMKLAKKLGELDPLMKPKQDRYALRT    357
Sobic.004G220500      GKPEYTDHLTHKLKHHPGQIEAAAIMEHILEGSSYMKLAKKLGELDPLMKPKQDRYALRT    353
Sobic.004G220600      GKPEYTDHLTHKLKHHPGQIEAAAIMEHILEGSSYMKLAKKLGELDPLMKPKQDRYALRT    353
Sobic.004G220700      GKPEYTDHLTHKLKHHPGQIEAAAIMEHILEGSSYMKLAKKLGELDPLMKPKQDRYALRT    353
Sobic.004G220300      GKPEYTDHLTHKLKHHPGSIEAAAIMEHILDGSAFMKHAKKVNELDPLLKPKQDRYALRT    342
Sobic.006G148800      GKPEYTDHLTHKLKHHPGSIESAAIMEHILDGSSFMKHAKEVNAMDPLLKPKQDRYALRT    342
                      *:**:**** * ******.**:******:*:**::*  *::   :*** ********:**

Sobic.001G160500      TPQWLGPQVEVIRFATKSIEREINSVNDNPVIDVARGKALHGGNFQGTPVGVSMDNTRLA    416
Sobic.006G148900      SPQWLGPQIEVIRAATKSIEREINSVNDNPLIDVSRGKALHGGNFQGTPIGVSMDNTRLA    413
Sobic.004G220400      SPQWLGPQIEVIRFATKSIEREINSVNDNPLIDVSRGKALHGGNFQGTPIGVSMDNTRLA    417
```

```
Sobic.004G220500      SPQWLGPQIEVIRAATKSIEREINSVNDNPLIDVARSKALHGGNFQGTPIGVSMDNTRLA    413
Sobic.004G220600      SPQWLGPQIEVIRAATKSIEREINSVNDNPLIDVARSKALHGGNFQGTPIGVSMDNTRLA    413
Sobic.004G220700      SPQWLGPQIEVIRAATKSIEREINSVNDNPLIDVARSKALHGGNFQGTPIGVSMDNTRLA    413
Sobic.004G220300      SPQWLGPQIEVLRAATKSIEREVNSVNDNPVIDVHRGKALHGGNFQGTPIGVSMDNARLA    402
Sobic.006G148800      SPQWLGPQIEVIRAATKSIEREVNSVNDNPVIDVHRGKALHGGNFQGTPIGVSMDNARLA    402
                      :*******:**:* ********:*******:*** *.************:******:***

Sobic.001G160500      VAAIGKLMFAQFTELVSSFYSNGLPSNLSGGRDPSLDYGFNGAEVAMASYCSELQFLANP    476
Sobic.006G148900      VAAIGKLMFAQFSELVNDFYNNGLPSNLSGGRNPSLDYGFKGAEIAMASYCSELQFLANP    473
Sobic.004G220400      LAAIGKLMFAQFSELVNDYYNNGLPSNLSGGRNPSLDYGFKGAEIAMASYCSELQFLGNP    477
Sobic.004G220500      IAAIGKLMFAQFSELVNDYYNNGLPSNLSGGRNPSLDYGFKGAEIAMASYCSELQFLGNP    473
Sobic.004G220600      IAAIGKLMFAQFSELVNDYYNNGLPSNLSGGRNPSLDYGFKGAEIAMASYCSELQFLGNP    473
Sobic.004G220700      IAAIGKLMFAQFSELVNDYYNNGLPSNLSGGRNPSLDYGFKGAEIAMASYCSELQFLGNP    473
Sobic.004G220300      IANIGKLMFAQFSELVNEFYNNGLTSNLAGSRNPSLDYGFKGTEIAMASYCSELQYLGNP    462
Sobic.006G148800      IANIGKLMFAQFSELVNEFYNNGLTSNLAGSRNPSLDYGFKGTEIAMASYCSELQYLANP    462
                      :* *********:***..:*.*** ***:*.*:*******:*:*:*********:*.**

Sobic.001G160500      VTNHVQTAEQHNQSVNSLGLISSRMTAEAVTILKLMSSTFLIALCQAIDLRQLEESIKAA    536
Sobic.006G148900      VTNHVQSAEQHNQDVNSLGLISSRKTAEAVDVLKLMSSTFLIALCQAVDLRHLEENLKSA    533
Sobic.004G220400      VTNHVQSAEQHNQDVNSLGLISSRKTAEAIDILKLMTSTFLIALCQAIDLRHIEENVKAA    537
Sobic.004G220500      VTNHVQSAEQHNQDVNSLGLISSRKTAEAIEILKLMSSTFLIALCQAVDLRHIEENVKSA    533
Sobic.004G220600      VTNHVQSAEQHNQDVNSLGLISSRKTAEAIEILKLMSSTFLIALCQAVDLRHIEENVKSA    533
Sobic.004G220700      VTNHVQSAEQHNQDVNSLGLISSRKTAEAIEILKLMSSTFLIALCQAVDLRHIEENVKSA    533
Sobic.004G220300      ITNHVQSAEQHNQDVNSLGLVSARKTAEAIDILKLMSSTYIVALCQAIDLRHLEENIKTS    522
Sobic.006G148800      ITNHVQSAEQHNQDVNSLGLVSARKTAEAVDILKLMSSTYMVALCQAVDLRHLEENLKSA    522
                      :*****:******.******:*:* ****: :****:**:::*****:***::**.:*::

Sobic.001G160500      VKKCVARVAKKCLAMDDDAAA-----AAAQLLGAVDRVAVFTYAEDPCRPSLPLMQKLRV    591
Sobic.006G148900      VKSCVTTVAKKTLSTSATGGLHNARFCEKDLLTAIDREAVFAYADDPCSANYPLMQKMRS    593
Sobic.004G220400      VKNCVTQVAKKSLSLNARGGLHNARFCEKDLQTAIDREAVFAYADDPCSPNYPLMQKLRA    597
Sobic.004G220500      VKSCVMTVAKKTLSTNSTGGLHVARFCEKDLLQEIEREAVFAYADDPCSANYPLMKKLRN    593
Sobic.004G220600      VKSCVMTVAKKTLSTNSTGGLHVARFCEKDLLQEIEREAVFAYADDPCSANYPLMKKLRN    593
Sobic.004G220700      VKSCVMTVAKKTLSTNSTGGLHVARFCEKDLLQEIEREAVFAYADDPCSANYPLMKKLRN    593
Sobic.004G220300      VKNTVTQVAKKVLTMNPSGDLSSARFSEKELITAIDREGVFTYAEDPASASLPLMTKLRA    582
Sobic.006G148800      VKNCVMAAARKVLTTSLDGDLHSARFSEKALLTAIDREAVYGYYDDPCSANSPLMKKIRA    582
                      **. *  .*:* *: .  .       .   *   ::* .*: * :**.  . *** *:*

Sobic.001G160500      VLMEHALGAAEGAV-ETTSILAKVAEFEQQLRAVLPDEVEAARAAVESGTA--PKRIAEC    648
Sobic.006G148900      VLVEHALANGEAERDPETSVFAKVATFEEELRAALPREVDAARAAVENGTAAVPNRIAEC    653
Sobic.004G220400      VLIEHALANGDAERVVETSIFAKVAEFEQQVRAALPKEVEAARAAVESGNPLVPNRIKEC    657
Sobic.004G220500      VLVERALANGAAEFDAETSVFAKVAQFEEELRAALPKAVEAARAAVENGTAAIPNRITEC    653
Sobic.004G220600      VLVERALANGAAEFDAETSVFAKVAQFEEELRAALPKAVEAARAAVENGTAAIPNRITEC    653
Sobic.004G220700      VLVERALANGAAEFDAETSVFAKVAQFEEELRAALPKAVEAARAAVENGTAAIPNRITEC    653
Sobic.004G220300      VLVDHALSSGDAERE--PSVFSKITKFEEELRAVLPREVEAARVAVAEGTAPVANRIADS    640
Sobic.006G148800      VLVDHALASGEAEKDASASVFSKINRFEEALREALPREMEAARVAFETGTAPIGNRIKDS    642
                      **:::**. . .      *:::*:  **: :* .**  ::***.*.  *.    :** :.

Sobic.001G160500      RSYPLYRFVREELGAEYLTGEKTRSPGEEVDKVVIAMNQRKHIDPLLECLSDWKGAPLHA    708
Sobic.006G148900      RSYPLYRFVREELGTEYLTGEKTRSPGEEVNKVFVAMNLGKHIDAVLECLKEWNGEPLPI    713
Sobic.004G220400      RSYPLYRFVREEVGTQYLTGEKTRSPGEELNKVLVAINQRKHVDPLLECLKEWNGEPLPL    717
Sobic.004G220500      RSYPLYRFVRQEVGAVYLTGEKTRSPGEELNKVLVAINQGKHIDPLLECLKEWNGEPLPI    713
Sobic.004G220600      RSYPLYRFVRQEVGAVYLTGEKTRSPGEELNKVLVAINQGKHIDPLLECLKEWNGEPLPI    713
Sobic.004G220700      RSYPLYRFVRQEVGAVYLTGEKTRSPGEELNKVLVAINQGKHIDPLLECLKEWNGEPLPI    713
Sobic.004G220300      RSFPLYRFVREELGCVFLTGEKLKSPGEECTKVFNGINQGKLVDPMLECLKEWDGKPLPI    700
Sobic.006G148800      RSYPLYRFIRQDLGAVYLTGEKLKSPGEECNKVFLALSEGKLIDPMLECLKEWDGKPLPI    702
                      **:*****:*:::*  :***** :*****  **. .:.  * :* :****.:*.* **

Sobic.001G160500      T*---  709
Sobic.006G148900      C*---  714
Sobic.004G220400      C*---  718
Sobic.004G220500      C*---  714
Sobic.004G220600      C*---  714
Sobic.004G220700      C*---  714
Sobic.004G220300      NVVN*  704
Sobic.006G148800      C*---  703
```

Supplementary Table S2. List of *cis*-elements identified in promoter region of PAL genes in sorghum

***Cis*-elements**	**SbPAL1**	**SbPAL2**	**SbPAL3**	**SbPAL4**	**SbPAL5**	**SbPAL6**	**SbPAL7**	**SbPAL8**
3-AF1 binding site	0	0	1	1	0	0	0	0
A-box	2	1	0	0	2	0	1	1
AAGAA-motif	0	1	1	1	2	2	1	0
ABRE	2	10	1	1	5	2	5	8
ABRE3a	1	1	0	0	2	0	1	1
ABRE4	1	1	0	0	0	0	1	1
AC-I	0	1	0	0	0	0	3	2
AC-II	0	1	0	0	2	0	1	2
ACE	0	1	0	0	0	0	0	1
AE-box	0	1	0	0	1	0	0	0
ATC-motif	0	1	0	0	0	0	0	0
ARE	2	0	4	1	0	0	7	2
AT~ABRE	0	0	0	0	0	0	0	1
AT1-motif	0	0	1	1	0	0	0	0
AT~TATA-box	1		2	2	2	3	1	0
AuxRE	0	0	0	0	0	0	0	1
Box 4	0	0	1	1	0	0	4	0
CAAT-box	28	16	26	26	18	41	26	40
CARE	0	0	0	0	0	0	1	0
CAT-box	1	3	1	1	1	0	3	0
CCAAT-box	0	0	1	1	0	1	1	0
CCGTCC motif	2	1	0	0	2	0	1	1
CCGTCC-box	1	1	0	0	2	0	1	1
CGTCA-motif	3	2	0	0	3	0	2	0
DRE core	1	1	0	0	1	2	0	1
DRE1	1	1	1	1	0	0	0	0
ERE	0	1	1	1	1	1	0	0
F-box	1	0	0	0	0	0	3	0
G-Box	0	0	0	0	1	0	1	0
G-box	3	8	1	1	3	1	0	4
GARE-motif	1	0	2	2	0	0	1	0
GATA-motif	0	0	0	0	0	2	1	0
GC-motif	2	2	0	0	3	2	1	1
GC-repeat	0	1	0	0	0	0	0	0
GT1-motif	1	0	0	0	2	1	0	3
HD-Zip 1	1	0	0	0	0	0	0	0

I-box	0	0	0	0	2	0	0	0
LAMP-element	1	0	1	1	0	0	0	0
LTR	1	0	2	2	0	0	0	1
MBS	1	0	0	0	0	1	1	1
MRE	0	0	0	0	1	0	0	0
MSA-like	1	0	1	1	0	0	0	0
MYB	6	3	4	4	4	4	3	5
MYC	4	1	5	5	5	1	2	3
Myb	1	0	0	0	0	1	0	2
Myb-binding site	2	2	2	2	2	0	0	0
MYB recognition site	0	0	1	0	0	1	1	0
MYB-like sequence	0	0	0	0	0	2	1	2
Myc	1	0	0	0	0	0	0	0
O2-site	0	2	1	1	1	2	2	0
OCT	1	0	0	0	0	0	0	0
P-box	0	2	1	1	0	0	0	0
RY-element	0	0	0	0	0	4	0	0
STRE	2	3	1	1	5	2	1	6
Sp1	4	4	1	1	4	0	1	1
TATA	0	0	0	0	1	0	0	0
TATA-box	18	13	14	14	16	17	15	19
TATC-box	1	0	0	0	0	0	0	1
TC-rich repeats	1	0	0	0	0	0	0	0
TCA	0	0	1	1	0	0	0	0
TCCC-motif	0	0	0	0	0	1	2	1
TCT-motif	2	1	1	1	0	0	0	0
TGA-element	0	0	1	1	1	1	1	1
TGACG-motif	3	2	0	0	3	1	2	0
W box	0	1	0	0	2	0	0	2
WRE3	3	1	1	1	1	1	3	3
WUN-motif	0	0	0	0	0	0	1	0
as-1	3	2	0	0	3	1	2	0
chs-CMA2b	0	1	0	0	0	0	0	1
motif I	0	0	0	0	1	0	0	0
box S	0	0	0	0	1	0	0	0
dOCT	0	0	0	0	0	0	0	2

Supplementary Table S3. Primer information used in this experiment

Gene I.D.	Gene name	Primer name	Primer sequence (3'-5')
Sobic.004G220300	SbPAL1	PAL1-F	GCCCTCCGTGTTCTCCAAGATCAC
		PAL1-R	TTCGCCACGGGGGCGGTGCCCTC
Sobic.004G220400	SbPAL2	PAL2-F	CTCCCATTGGTGTGTCCAT
		PAL2-R	AAGGCCGTTGTTGTAGTAGTC
Sobic.004G220500	SbPAL3	PAL3-F	GGTGCTCGTTGCCATCA
		PAL3-R	TCTCTCTCTCTATCCGCTTCTC
Sobic.004G220700	SbPAL5	PAL5-F	GGTGCTCGTTGCCATCA
		PAL5-F	ACAGTCTTCTGCTCCTTGTATTT
Sobic.001G160500	SbPAL6	PAL6-F	GAAGCTCATGTCCTCCACTTT
		PAL6-F	CGTCCATGGCCAGACATT
Sobic. 006G148800	SbPAL7	PAL7-F	AGGCCGAGAAGGACGCCAGCGCG
		PAL7-R	TTAGCAGATGGGCAGGGGCTTGC
Sobic.006G148900	SbPAL8	PAL8-F	GTGTTCTGCGAGGTGATGAA
		PAL8-R	CTTCTTCGCGAGCATCATGTA
Sobic.002g350400	Tubulin	Tub-F	GAGGTGACGATGCTTTCAACAC
		Tub-R	CACAGGTCAACAATCTCCTTGC
